# Factors associated with postpartum depression among adolescents in Uganda

**DOI:** 10.11604/pamj.2018.30.170.15333

**Published:** 2018-06-25

**Authors:** Catherine Atuhaire, Samuel Nambile Cumber

**Affiliations:** 1 Department of Nursing, Faculty of Medicine, Mbarara University of Science and Technology, Mbarara, Uganda; 2 Institute of Medicine, Department of Public Health and Community Medicine, University of Gothenburg, Box 414, SE-405 30 Gothenburg, Sweden

**Keywords:** Postpartum, depression, risk factors, adolescents, Uganda

## Abstract

Postpartum depression (PPD) is a common and disabling public health complication of the postpartum period in women. It is believed to occur three times more commonly in developing countries than in the developed world and is more prevalent among women in the first six weeks after birth. Research suggests that postpartum depression is more commonly diagnosed among adolescents and may be a risk factor for poor growth and development in children born to these mothers. Therefore, adolescents are a special age group that requires specific health care maternal interventions in order to detect and treat post-partum depression.

## Introduction

The postpartum period is associated with an increased risk for the development of maternal depression. Maternal depression is a common and debilitating mental disorder which has become of significant public health concern, most especially in the least developed countries [1]. These depressive disorders are classified into three categories based on severity and time of onset after child birth [2]. The baby or maternal blues occur in about 40 to 80 percent in mothers during the first postpartum month, and are usually mild, self-limiting and with little consequence to the health of the mother and child [3]. But, if it is not managed appropriately, the mother is at increased risk of suffering from postpartum depression. Postpartum depression has a prevalence of 13 to 19 percent [4] and if still not treated, the mother is likely to suffer from postpartum psychosis. Postpartum Depression (PPD) is a non-psychotic depressive episode which occurs in women from 4 weeks to 6months following child birth [2]. Adolescents are a special age group that requires specific health care maternal interventions [5]. Approximately, 1.2 billion adolescents are aged between 10-19 years of age worldwide of which up to 16% constitute the world's population [6]. The majority (86%) of these adolescents live in the developing countries. By the time they are 19 years old, half of adolescent girls in developing countries are sexually active, about 40% are married and close to 20% have children [7]. In Uganda, there are 34.6 million people of which 34.8% are adolescents [8]. Since 20I0, the number of adolescent girls who get pregnant before the consent age of 18 years has increased by 25 percent in Uganda. While there is clear evidence of affecting child morbidity and mortality, the factors associated with PPD in adolescence are inconclusive and limited [9]. Impeded maternal care poses a high risk in the first year of life because infants require more care during this period and are more susceptible to their mothers' depressive moods [10, 11]. Uganda and other developing countries maternal mental health is still largely neglected [4]. Consequently, little is known about the risks associated with postpartum depression among adolescents in Uganda since it has not been prioritized and diagnosed in Ugandan hospitals.

**Aim of study**: The aim of the study was to assess the manifestations and risk factors associated with postpartum depression among adolescents in Uganda.

**Objectives**: The magnitude of PPD in Uganda is under documented. This explains the motivation of this paper, whose objectives are to assess the risk factors of postpartum depression among adolescents in Uganda.

## Methods

In this short communication study, 16 data base were used during the search for articles. The data base used were: PubMed, EBSCO, ECO, DIVA, TOXNET, LIBRIS, Web of Science, ArticleFirst, PsycINFO, Africa Bibliography, Bibliography of Africana periodical Literature, African Journal On-Line, Education Resources Information Centre, Google Scholar, GenderWatch and SwePub. The above 16 data bases were used to search for articles, journals and reports. From the literature search, 21 citations were found, using keywords such as Postpartum, depression, risk factors, adolescents, teenagers, Uganda. The paper focused on Postpartum depression (PPD) in Uganda. And for this reason, many of the literature found during the search was eliminated and only 4 articles met the interior criteria and were those 4 used to answer the specific objectives.

## Results

UDHS, 2016 reports a 1 percent increase in teenage pregnancy from 24 to 25 percent in 2016 meaning that every 3 in 10 adolescents have begun child bearing [7]. The risk factors for development of PPD are similar in both developed and developing countries, but mothers in Uganda are at higher risk [12]. A study carried out in an urban setting of Uganda which is evidenced by the participants' level of education that was secondary school reported that postpartum depression was significantly associated with young mothers (teenagers), mothers having unplanned pregnancies while they were single parents, congenital abnormalities in the baby, not preferred sex of the child and negative life occurrences before child birth and mother's illness [12]. Another study carried out in Uganda reported that postpartum depression was common among HIV/AIDS infected and affected mothers, unexpected death of a child or spouse, poverty most especially those in the rural setting and violence abuse among partners [13]. A similar study carried out among married adults in rural Uganda noted that polygamous spouses, marital problems, inability of the infant to breast feed, high parity and spouse support provided to the mother following child birth had statistical significant relationship with postpartum depression [14] (Table 1).

**Table 1:** Articles used to answer the research objectives of factors associated with postpartum depression in adolescents

No.	Author names	Topic	Year	Country Uganda	Aim	Results
1.	UBOS	Uganda Demographic and Health survey	2012	Uganda	The burden of teenage pregnancies	There is an increase in teenage pregnancies
2.	*JN Nakku, G Nakasi, F Mirembe*	Postpartum major depression at six weeks in primary health care: prevalence and associated factors	2006	Uganda	Assessing the prevalences and risk factors of postpartum depression	Prevalence of 6.1% and psychiatric disorder was significantly associated with young age, being single, negative life events, unplanned pregnancy, unwanted sex of baby and current physical illness in both mother and newborn
3.	Kaida A, Matthews LT, Ashaba S, Tsai AC, Kanters S, Robak M	Depression during pregnancy and the postpartum among HIV-infected women on Antiretroviral Therapy in Uganda	1999	Uganda	Assess for depression from pregnancy through the postpartum period among HIV women on ART	Increasing time on ART, viral suppression, better physical health, and “never married” were independently associated with lower mean depression scores
4.	Kakyo TA, Muliira JK, Mbalinda SN, Kizza IB, Muliira RS	Factors associated with depressive symptoms among postpartum mothers in a rural district in Uganda	2012	Uganda	To assess the factors associated with depressive symptoms in postpartum mothers in rural Uganda	Statistically significant relationships were found between PDS and factors such as number of female sexual partners the husband has’ current problems in marriage, participant's parity, infant's ability to breast feed and husband support during the postpartum period

## Discussion

The increase in teenage pregnancies has been partly attributed to the low level of education with at least three in every 10 of them having no education. 78% of adolescents are currently attending formal education and 22% are school drop outs before secondary level [7]. One in four adolescent girls aged 15-19 have had a child or are pregnant in Uganda but 42% of all adolescent pregnancies are unintended [15]. Unintended pregnancies are associated with adverse health effects like postpartum depression that inflict considerable burden to Ugandan economies. Adolescent mothers undergo a great deal of challenges as young mothers which later impacts their functioning during the postpartum period. These mothers suffer from an increased rate of postpartum depression symptoms [16]. In adolescents, postpartum depression is characterized by feelings of loss and sadness and, sometimes self-esteem is lost [17].

In addition, mothers may manifest with depressed mood, loss of energy, self-guilt, suicidal ideas. These adolescents sometimes have less concentration, are restless and agitated, irritable, anxious, unworthiness feelings, have disturbed sleep, and tearfulness [12]. In case of physical symptoms, these patients may present with chest pain, numbness, headaches and hyperventilation [4]. The factors associated with postpartum depression were found to be related to those in South Asia and SSA [18]. High parity has been implicated as a major contributing factor to PPD [19] found many associated factors although other countries have shown mixed evidence for example in the UAE, it is a protective factor for PPD, while in low-income countries like Nepal and Pakistan, high parity increases family stress and the risk of PPD [20]. This could be attributed to the physical and financial burden. In most Ugandan cultures, there is gender bias in favor of men who are dominating and looked at as future leaders. For pregnant women to be accepted in society, it has been discovered that majority expressed a strong desire to know the fetal sex at ultrasound [21, 22].

This desire may be attributed to societal pressure. Studies in other countries like India show that the preference for male children is deeply rooted in Indian society. Thus, women who already have a female child face greater stress because of their wish that their new infant be a boy. In the event that the child is a girl, the risk of depression is greater [23]. Recently, a study by [24] revealed that untreated postpartum depression affects the health of the mother, infant and family. It was estimated that reductions in the prevalence of postpartum depression could lead to a reduction in impaired child growth and development by up to 30 percent. Data from developing countries suggest that maternal depression may be a risk factor for poor growth and development in young children [11]. PPD compromises child health outcomes [25] and has been associated with disturbances in the mother-infant relationship, bonding and in the child's cognitive development.

## Conclusion

Postpartum depression affects women in both developed and undeveloped countries and the adolescent mothers are more at risk of suffering from it. The risk factors are similar except that the underdeveloped countries in particular sub-Saharan African countries like Uganda experience a greater burden of the risk factors such as the age of the mothers, HIV/AIDS, low social economic status, and culture. Normally, the birth of a newborn baby is considered a joyful event and so mothers maybe embarrassed to express their depressive feelings and this too can be a barrier to early detection and treatment of postpartum depression.

**Recommendations:** Government of Uganda needs to consider postpartum depression as a public health problem that is neglected among adolescent mothers. Most mothers are reluctant to express their feelings and during the postnatal clinic visits, the health care givers put much focus on the baby for immunization services and no attention is paid to the mother at all. This leaves postpartum depression to go undetected. Early screening, treatment of postpartum depression and provision of psychosocial support should be part of routine perinatal care and should be considered as an integral component of maternal postpartum care in Uganda.

### What is known about this topic

- Postpartum depression is more prevalent among adolescents;
- Adolescents are likely to be more affected with the PPD in terms of prevalence and frequency as compared to the adults;
- Patients suffering from postpartum depression have similar clinical manifestations but the associated risk factors differ between the developed and developing countries.

### What this study adds

- Findings will provide a basis for health workers screening and treating PPD, this in turn will improve maternal infant bond and also promote good health of the mother and child;
- The findings from the study will highlight the manifestations and risk factors associated with PPD that nurses could base on to develop strategies to address it; this will contribute towards prevention of PPD;
- The findings will stimulate more research to be done in Uganda and possibly be a basis for informed policy formulation.

## Competing interests

The author declare no competing interests.
